# Exploring Shared Susceptibility between Two Neural Crest Cells Originating Conditions: Neuroblastoma and Congenital Heart Disease

**DOI:** 10.3390/genes10090663

**Published:** 2019-08-30

**Authors:** Alessandro Testori, Vito A. Lasorsa, Flora Cimmino, Sueva Cantalupo, Antonella Cardinale, Marianna Avitabile, Giuseppe Limongelli, Maria Giovanna Russo, Sharon Diskin, John Maris, Marcella Devoto, Bernard Keavney, Heather J. Cordell, Achille Iolascon, Mario Capasso

**Affiliations:** 1 Dipartimento di Medicina Molecolare e Biotecnologie Mediche, Università degli Studi di Napoli Federico II, 80131 Naples, Italy; 2 CEINGE Biotecnologie Avanzate, 80145 Naples, Italy; 3 IRCCS SDN, Istituto di Ricerca Diagnostica e Nucleare, 80143 Naples, Italy; 4 Division of Cardiology, Università degli Studi della Campania “Luigi Vanvitelli” - AO dei Colli, Presidio Monaldi, 80121 Naples, Italy; 5 Division of Oncology and Center for Childhood Cancer Research, The Children’s Hospital of Philadelphia, Philadelphia, PA 19104, USA; 6 Department of Pediatrics, The Perelman School of Medicine, University of Pennsylvania, Philadelphia, PA 19104, USA; 7 Division of Genetics, The Children’s Hospital of Philadelphia, Philadelphia, PA 19104, USA; 8 Department of Translational and Precision Medicine, University of Rome “La Sapienza”, 00185 Rome, Italy; 9 Department of Biostatistics, Epidemiology and Informatics, Perelman School of Medicine, University of Pennsylvania, Philadelphia, PA 19104, USA; 10 Division of Cardiovascular Sciences, School of Medical Sciences, Faculty of Biology, Medicine and Health, University of Manchester, Manchester M13 9PL, UK; 11 Manchester University NHS Foundation Trust, Manchester Academic Health Science Centre, Manchester M20 4BX, UK; 12 Institute of Genetic Medicine, Newcastle University, Central Parkway, Newcastle upon Tyne NE1 3BZ, UK

**Keywords:** genome wide association studies, neuroblastoma, congenital heart disease

## Abstract

In the past years, genome wide association studies (GWAS) have provided evidence that inter-individual susceptibility to diverse pathological conditions can reveal a common genetic architecture. Through the analysis of congenital heart disease (CHD) and neuroblastoma (NB) GWAS data, we aimed to dissect the genetic susceptibility shared between these conditions, which are known to arise from neural crest cell (NCC) migration or development abnormalities, via identification and functional characterization of common regions of association. Two loci (2q35 and 3q25.32) harbor single nucleotide polymorphisms (SNPs) that are associated at a *p*-value < 10^−3^ with conotruncal malformations and ventricular septal defect respectively, as well as with NB. In addition, the lead SNP in 4p16.2 for atrial septal defect and the lead SNP in 3q25.32 for tetralogy of Fallot are less than 250 Kb distant from the lead SNPs for NB at the same genomic regions. Some of these shared susceptibility loci regulate the expression of relevant genes involved in NCC formation and developmental processes (such as *BARD1,* *MSX1,* and *SHOX2*) and are enriched in several epigenetic markers from NB and fetal heart cell lines. Although the clinical correlation between NB and CHD is unclear, our exploration of a possible common genetic basis between NB and a subset of cardiac malformations can help shed light on their shared embryological origin and pathogenetic mechanisms.

## 1. Introduction

Neuroblastoma (NB) is an embryonic tumor arising from the sympathetic nervous tissue and is among the most frequent cancers diagnosed in early infants, accounting for 13% of all deaths due to childhood malignancies [1]. Its etiology is due to an overgrowth in the sympathetic ganglion where neural crest derived progenitors reside. Whereas familial NB is rare [2], sporadic NB has a higher incidence: The study of its genetic susceptibility can therefore benefit from a more abundant cohort of patients and has thus been largely investigated by means of genome wide association studies (GWAS) [3,4] and candidate gene approaches [5,6,7].

Congenital heart disease (CHD) is one of the most frequent inborn disorders in infants, affecting 7 in 1000 live births and is a major cause of childhood death and long term morbidity [8]. Complex genetic mechanisms underlie cardiac development and its anomalies, and a number of different defects could be the cause—such as migration defects, reduced specification or overproduction of neural crest-derived mesenchymal cell types—and efforts have been made to try and elucidate causative variants affecting these conditions [9,10,11,12].

Neural crest cells (NCC) development and migration abnormalities have been conjectured to be implicated in the genesis of both CHD and NB [13,14,15,16], and there are case reports in the literature of patients affected with both of these conditions simultaneously [17]. George and colleagues [18] demonstrated that children affected with NB have a higher prevalence of CHD; however, van Engelen and colleagues [19] have denied evidence of association between these two conditions. A review of more than 1900 cases showed that NBs account for approximately 17% of the malignancies seen in Costello and Noonan syndromes [20], a disorder characterized by diverse tissue and organ defects, including CHD [21]. Lombardo and colleagues [22] very recently reported an association between CHD and mutations in *PHOX2B*, a susceptibility gene for familial NB [23]. In spite of some negative evidence, it is possible that NB and CHD share susceptibility loci but that their phenotypes are not highly penetrant in individuals with certain susceptible mutations.

Demonstrating a correlation between NB and CHD could provide useful information to patients suffering from these conditions, including the opportunity of specific genetic counseling addressing the possible onset of the other disease. Although epidemiological studies are a powerful tool for addressing this question, genome wide association studies (GWAS) can provide a deeper level of understanding of the genetics underlying phenotypic traits, including pathological conditions. The accumulation of large-scale genomic datasets has led to the detection of novel loci associated with diverse traits and enhanced the study of shared genetic factors across phenotypes, but a thorough characterization of these identified loci would be advisable, both at the genetic and epigenetic level. Using data from different conditions can reveal the presence of common genetic risk factors and shared causal pathways, thus improving our understanding of disease.

Given NB and CHD common embryological derivation from NCC [21,24], we analyzed GWAS results for these traits in order to evaluate the extent of shared genetics between NB and seven CHD conditions: atrial septal defect/patent foramen ovale (ASD/PFO), conotruncal malformations (CM), double outlet right ventricle (DORV), left-sided malformations (LH), transposition of the great arteries (TGA), tetralogy of Fallot (ToF), and ventricular septal defect (VSD).

## 2. Materials and Methods

### 2.1. Neuroblastoma GWAS Summary Statistics

GWAS summary statistics were taken from the work of McDaniel and colleagues [25]. These refer to a European-American cohort of 2101 cases and 4202 matched controls (Table 1) assayed with Illumina HumanHap550 v1, HumanHap550 v3, and Human610 single nucleotide polymorphism (SNP) arrays. Genotype phasing was performed using SHAPEIT v2.r790 [26] and data from 1000 Genomes Phase 1 Release 3. Subsequent imputation was performed genome-wide using IMPUTE2 v2.3.1 [27] for all SNPs and indel variants annotated in 1000 Genomes Phase 1 Release 3. Only SNPs with minor allele frequency (MAF) >0.01 and info score >0.8 were considered. Manhattan plot of the NB GWAS is available in Figure S1 and characteristics of patients are summarized in Table S1.

### 2.2. CHD Genotypes

Genotypes from 5159 controls and patients with seven different subtypes of CHD, namely atrial septal defect/patent foramen ovale (ASD/PFO, 340 cases), conotruncal malformations (CM, 151 cases), double outlet right ventricle (DORV, 96 cases), left-sided malformations (LH, 387 cases), transposition of the great arteries (TGA, 207 cases), tetralogy of Fallot (ToF, 835 cases), and ventricular septal defect (VSD, 191 cases), were those included in the work of Cordell and colleagues [9]. Manhattan plots of the CHD GWAS are available in Figure S1.

### 2.3. CHD Genotypes Imputation

We used the Michigan Imputation Server [28] to perform imputation on the CHD datasets (reference panel: 1000G Phase 3 v5).

### 2.4. CHD Association Analysis

Dosage vcf files from the imputation output were fed to SNPTEST v2.5.4 beta1 [29] software and frequentist association test was used to compute summary statistics.

### 2.5. Evaluation of the Extent of Shared Genetics

**Figure 1:**
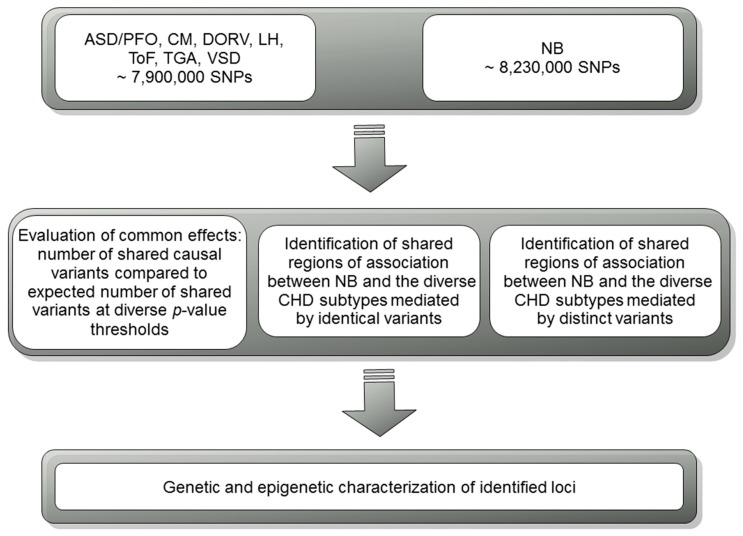
Study design and workflow.

The first step of our workflow (Figure 1) was the evaluation of the genome-wide shared genetics between NB and CHD. To this end, we pruned the whole set of common SNPs based on linkage disequilibrium (LD) with plink v1.9 [30] using a r^2^ threshold of 0.2 (plink --indep-pairwise function; parameters used are: r^2^ = 0.2; window size = 500 Kb; step size = 20) in order to consider only SNPs in approximate linkage equilibrium and evaluated the number of independent SNPs with association *p*-values above and below several thresholds (0.05, 0.01, 0.005, 0.001, 0.0005, 0.0001) in NB and in each CHD condition. This procedure prevents the overestimation of association signals due to LD structure (such as when multiple signals are present from high LD regions). If the two conditions do not share a genetic basis, these values should not deviate from random expectation. A 2 × 2 table for each *p*-value cutoff was created and one-sided Fisher exact tests were used as the statistical measure of significance and strength of association. We also ran simulations to assess the validity of our results: For each CHD condition, each SNP was randomly assigned a *p*-value from the list of observed *p*-values deriving from the association analysis for that condition. We repeated this a thousand times, and an empirical *p*-value was calculated from the proportion of simulations having a number of SNPs below a given *p*-value threshold in both datasets (NB and one CHD type) greater than or equal to the observed number of SNPs below the same *p*-value threshold in both real datasets (NB and one CHD type) under consideration.

### 2.6. Identification of Colocalizing Association Signals

To identify shared association signals between NB and CHD, SNPs with an association *p*-value < 10^−3^ [31] in both NB and at least one CHD dataset were selected, and shared association signals were defined as those regions containing at least 10 such SNPs within a distance of less than 100 Kb.

To identify possibly colocalizing but distinct association signals, we selected SNPs with an association *p*-value < 10^−5^ in NB or at least one CHD dataset, and candidate regions were identified if at least 10 SNPs within a distance of less than 100 Kb were present. The threshold of *p*-value < 10^−5^ was chosen as it corresponds to an average of 1 false-positive association per GWAS in European populations [32]. The distance between lead SNPs of these candidate regions in NB and in the CHD conditions was then used to evaluate these potential colocalization signals deriving from distinct variants.

We also ran simulations to assess the significance of these distinct, colocalizing signals by randomly reshuffling the location of the associated regions in our NB dataset while keeping their size fixed: An empirical *p*-value was calculated from the proportion of simulations having a number of regions less than 250 Kb distant between NB and CHD greater than or equal to the real datasets.

A method proposed by Pickrell and colleagues [33] was also used to detect colocalizing regions. The algorithm generates posterior probabilities through a Bayesian approach for the hypotheses that the region harbors one variant associated to both, to only one or to none of the traits, or that the region contains one variant associated only to one trait and another variant associated only to the other trait.

### 2.7. Enrichment of Epigenetic Signatures in Susceptibility Loci

Enrichment in epigenetic features of several cell types related to neural crest cells (NCC) and heart, and NCC derived tumors (NB and melanoma) was computed through R VSE package from CRAN [34]. Briefly, it creates a network from SNPs that accounts for LD structure and generates a null model by sampling SNPs from a comprehensive pool of tag SNPs, thus recreating the same LD clusters as in the real data, matching each associated variant set to a random variant set with the same characteristics. Haploreg [35] was also used to perform enhancer enrichment analysis on sets of variants (binomial test), using as background frequency the overlap from 1000 Genome variants with a frequency above 5% in any population.

### 2.8. eQTL Analysis

LinDA [36] was used to identify genes regulated by variants of interest, as well as tissues involved. This tool takes as input a list of variants and queries 199 datasets belonging to 53 projects, comprising 15 human populations and 33 body districts, resulting in 486,244 eQTLs and 36,768 eGenes.

## 3. Results

### 3.1. Evaluation of the Genome Wide Extent of Shared Genetic Association

**Figure 2:**
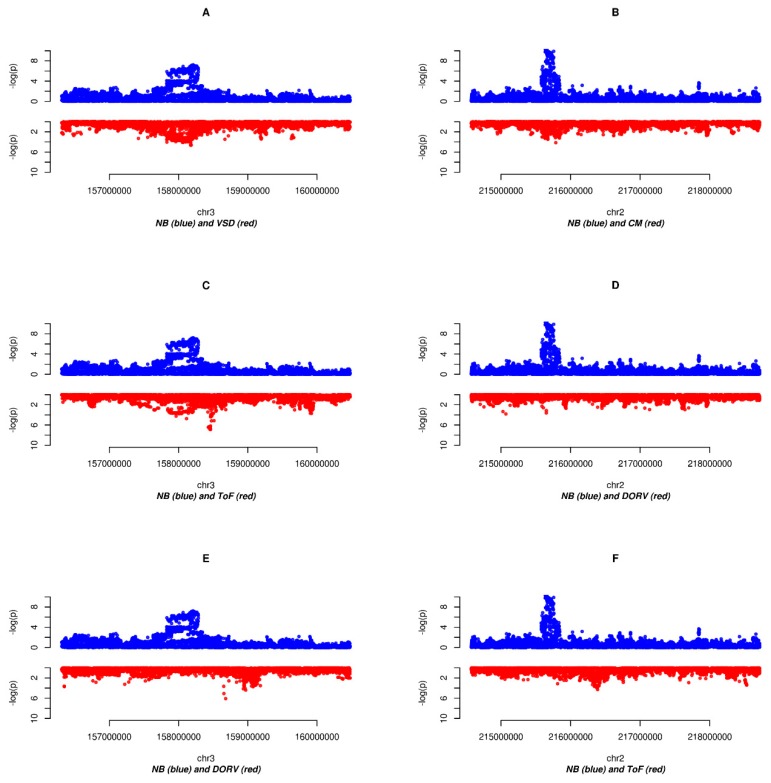
Regional association plots of significant loci described in text. In blue is NB, in red are different subtypes of CHD. (**A**) NB and VSD at 3q25.32, (**B**) NB and CM at 2q35, (**C**) NB and ToF at 3q25.32, (**D**) NB and DORV at 2q35, (**E**) NB and DORV at 3q25.32, (**F**) NB and ToF at 2q35.

To evaluate the genome-wide shared genetic signals between NB and CHD, we selected a subset of independent SNPs in approximate linkage equilibrium with each other and evaluated for each condition the number of SNPs with association *p*-value above and below different thresholds. We used Fisher exact test and simulation analysis to evaluate whether NB and each CHD condition in turn share more SNPs above and below the *p*-value thresholds than expected by chance [37] (see Materials and Methods Section 2.5). We found some evidence of shared association signals between NB and ASD/PFO, between NB and CM, and between NB and VSD (Table 2, Figure 2, Tables S2 and S3). As reported in Table 2, SNPs with *p*-value less than 0.01 are shared more frequently than expected between NB and all CHD datasets (Fisher exact test *p*-value = 0.02). Common association signals are also observed for low *p*-value thresholds when considering NB and CM (<0.005; Fisher exact *p*-value = 0.04) and when considering NB and VSD (<0.0005; Fisher exact *p*-value = 0.05) and for high *p*-value thresholds when considering NB and ASD/PFO (<0.05; Fisher exact *p*-value = 0.02).

### 3.2. Identification of Colocalizing Association Signals between NB and CHD

We defined shared association regions as genomic locations harboring at least 10 SNPs with association *p*-value < 10^−3^ in NB and in at least one CHD subtype (see Materials and Methods Section 2.6). With this procedure, we identified two main regions spanning over several Kb: one shared between NB and VSD (3q25.32; 399 SNPs, Figure 2A) and another one shared between NB and CM (2q35; 28 SNPs, Figure 2B). Two smaller regions were also identified: 12q21.31 has overlapping association signals in VSD, ASD/PFO, and NB and 14q24.3 has few SNPs which are significant both in NB and in ToF (Table 3). In this last case the direction of effect of the colocalizing SNPs in both datasets is the same, supporting a genuine shared allelic risk; whereas in the other cases the direction of effect is opposite, implying a shared genetic basis [38].

Following recent works that have pointed out the importance of effects mediated by distinct genetic determinants located in the same genomic regions for informing the causal relationship between different traits [33,39,40,41], we also evaluated evidence of this kind of spurious colocalization between NB and each CHD subtype. On the basis of the physical distance of lead SNPs in significant loci of association in NB and CHD we identified two colocalizing susceptibility regions: one region encompassing band 3q25, colocalizing between NB and ToF (Figure 2C and Table 4), and one further region in band 4p16.2 in NB and ASD/PFO (Figure S2 and Table 4, empirical *p*-values < 0.04 and <0.03, respectively). Table S4 reports all regions in the analyzed datasets with *p*-value < 10^−5^ and their relative distance.

**Figure 3:**
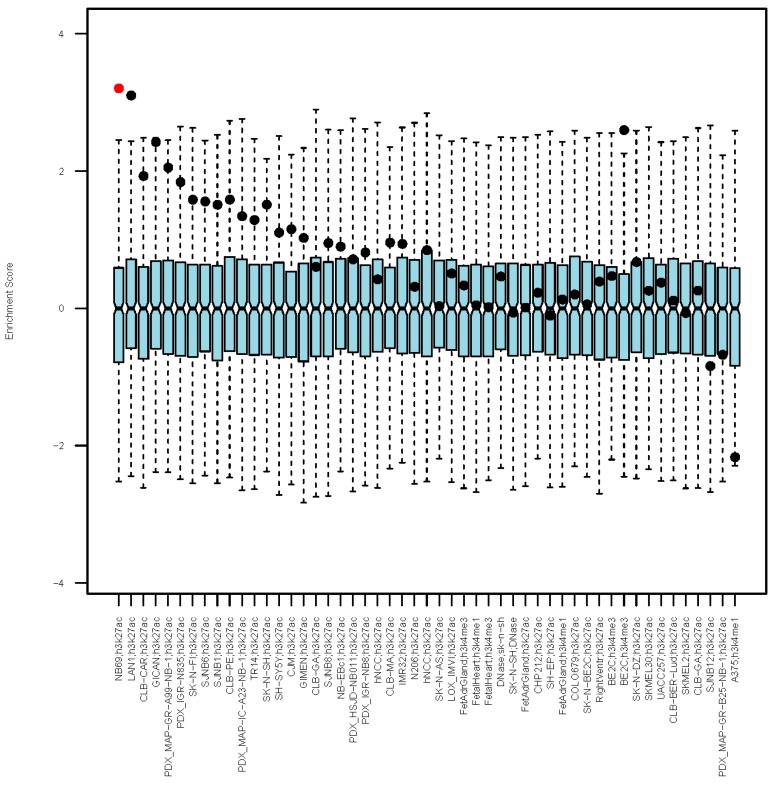
Box plots represent the distribution of overlap of the epigenetic feature under consideration with random sets of markers matched to the real set in terms of numerosity and LD structure. The bar inside each box corresponds to the median enrichment score of the null set. Whiskers span from minimum value to first quartile and from third quartile to maximum value. Dots represent the estimated enrichment in the real set of SNPs considered. One feature still significant after stringent multiple testing correction (Bonferroni corrected *p*-value < 0.01) is marked in red.

We also used a Bayesian method designed to test whether some genomic regions may harbor distinct variants associated to multiple traits [33] (see Materials and Methods Section 2.6). We found ten instances with a posterior probability >0.9 of containing distinct variants associated to NB and one or more CHD subsets (Table 5). Interestingly some of these identified regions were also identified through the other approaches although in different CHD subtypes: 2q35 has overlapping association signals between NB and CM (Figure 2B) and shows evidence of colocalization of NB with DORV and ToF (Figure 2D,F) and 3q25.32 has overlapping association signals between NB and VSD (Figure 2A) and shows evidence of colocalization of NB with DORV and ToF (Figure 2C,E). Region 4p16 contains both a signal of colocalization between NB and ASD/PFO (see above), as well as a signal of colocalization between NB and DORV. Two further colocalizing regions were identified: 6p22 (colocalizing NB with CM and NB with DORV) and 11p15 (colocalizing NB with DORV and NB with ToF) (Table 5 and Figure S2).

### 3.3. Enrichment in Epigenetic Markers in Colocalizing Regions

Epigenetic features overlapping genetic polymorphisms can help predict in which cell tissue that variant is likely to act [42]. Therefore we evaluated enrichment of several epigenetic markers from cell lines and tissues related to neural crest cells, NB, and heart development (see Table S5 for the complete list) in the set of the most significant SNPs previously identified (i.e., SNPs with *p*-value < 10^−3^ in NB and in at least one CHD subset in the regions reported in Table 3). In order to account for LD structure and prevent enrichment inflation in case of SNPs residing in high LD blocks, we used the Variant Set Enrichment (VSE) package from CRAN [34]. Results are shown in Figure 3. It can be seen that few NB cell lines (NB69, LAN1, BE2C) are significantly enriched in these regions. Interestingly (Table S6), it can be inferred that 2q35 is an epigenetic hotspot and has signatures from many cell lines whereas 3q25.32 has several epigenetic signatures from adrenal and fetal heart, which are also abundant in 2q35. The core 15-state model source for epigenomes in HaploReg [35] also gives evidence of enrichment in fetal heart signatures (*p*-value = 0.024) in these cross-associated variants.

### 3.4. Annotation of Colocalizing Regions

We used the genetic variants from the shared (association *p*-value < 10^−3^) and distinct (association *p*-value < 10^−5^) colocalizing signals and queried them for eQTL annotation through LinDA (http://linda.irgb.cnr.it/). Genes, variants, and tissues are listed in Table 6 and Table S7. This procedure allows to annotate genes from multiple catalogs that are likely regulated by the list of variants given as input in a tissue specific manner.

## 4. Discussion

The evaluation of shared association between epidemiologically linked conditions represents a powerful tool for the dissection of common and unique mechanisms in the development of phenotypic traits and the onset of pathological conditions [43,44]. On the basis of possible co-occurrence of NB and CHD [18,45] and their common derivation from NCC [21,24], we conducted a co-association study on these conditions, starting from a general evaluation of an excess of shared association signals, to a more detailed analysis of colocalizing association signals. We observed the strongest evidence of shared genetic architecture between NB and VSD, both at a genome-wide level (Table 2 and Figure 2) and at single loci (Table 3, Table 4 and Table 5), where in band 3q25.32 a region of nearly half Mb harbors 399 SNPs with association *p*-value below 10^−3^ in both conditions, which supports a genuine shared effect. This same region also shows evidence of shared association between NB and ToF and between NB and DORV.

Most of the SNPs that we identified in these loci of shared association show an opposite allelic effect. It was reported in the literature that for several conditions with a common pathological basis, shared genomic loci of association (such as the ones resulting from phenotype cross-trait analysis) show an opposite effect in several cases, possibly implying opposite functional changes in different cells/tissues affecting the same molecular trait or pathway [39].

Some of the regions detected by our colocalization analysis include intriguing candidate genes for NB and CHD. *MSX1* (4p16.2) is a homeobox gene involved in neural crest specification [46] that has been already identified as a CHD susceptibility gene [47]. Our results suggest that common variants can affect *MSX1* expression and can also predispose to NB. The role of *MSX1* in NB biology is also supported by a recent paper that demonstrates a signaling axis leading from *PHOX2B* via *MSX1* to Delta–Notch and proneural gene expression in NB pathogenesis [48]. Recently, NB has been diagnosed in a child with Wolf-Hirschhorn syndrome, a congenital disorder with characteristic facial features caused by microdeletion of the short arm of chromosome 4 encoding the *MSX1* gene [49].

Another relevant gene is *SHOX2* (3q25.32), a member of the homeobox family which is one of the major genes involved in the development of the sinoatrial node [50]; its proper function is of crucial relevance for the origin of arrhythmogenic heart disease [51]. Moreover, *SHOX2* is implicated in specifying neural systems involved in processing somatosensory information, as well as in face and body structure formation [52,53] and has been reported as involved in Cornelia de Lange syndrome—a condition that implies heart defects [52,54]. The relevance of this gene is supported from its association with eQTLs.

Our results and those from the literature show that the aforementioned genes are involved in developmental processes and that their abnormal functioning due to genetic alterations could predispose to the development of NB and CHD.

eQTL analysis points out the relevance of loci associated at 3q25.32; in fact 3 genes (*MLF1*, *RP11-538P18.2,* and *RSRC1*) are associated with variants relevant in at least four conditions: NB, DORV, ToF, and VSD. *MLF1* in particular has been recently described in NB [25] and seems to play an important role in tumorigenesis. *MLF1* is highly expressed in heart and has been identified as a novel modulator of cardiomyocyte proliferation [55]. Interestingly, our eQTL analysis using data from left ventricle tissues demonstrates that predisposing NB and CHD variants can affect *MLF1* expression.

We found that the known NB susceptibility gene *BARD1* (2q35) [4,56] lies in close proximity to a candidate susceptibility locus for CM; copy number alterations at the *BARD1* locus have been previously associated to developmental delay, coarctation of aorta and ToF [57], suggesting a role of *BARD1* in early organogenesis and heart formation.

The identification of regions of shared susceptibility can help in assigning a hierarchy in the pathogenic mechanisms of related conditions, and functional and epigenetic characterization of common associated SNPs from different traits can contribute to single out loci belonging to shared and unique pathways. Our results suggest a possible common genetic basis between these two NCC originating conditions. However, larger sample sizes and further studies will be needed to validate our results and better elucidate the shared genetic risk factors between NB and CHD.

## Figures and Tables

**Table 1:** Data sets used in this study.

Condition	Cases	Controls
ASD/PFO	340	5159
CM	151	5159
DORV	96	5159
LH	387	5159
NB	2101	4202
TGA	207	5159
ToF	835	5159
VSD	191	5159

Number of cases and controls for each dataset used. ASD/PFO: atrial septal defect/patent foramen ovale; CM: conotruncal malformations; DORV: double outlet right ventricle; LH: left-sided malformations; NB: neuroblastoma; TGA: transposition of the great arteries; ToF: tetralogy of Fallot; VSD: ventricular septal defect.

**Table 2:** Evaluation of the extent of shared genetic effects between neuroblastoma (NB) and congenital heart disease (CHD).

Dataset	*p*-value Threshold	Fisher Test *p*-value	Odds Ratio
ALL	0.0001	1	0
ALL	0.0005	1	0
ALL	0.001	1	0
ALL	0.005	0.92	0.46
ALL	0.01	0.02	1.56
ALL	0.05	0.84	0.94
ASD/PFO	0.0001	1	0
ASD/PFO	0.0005	1	0
ASD/PFO	0.001	1	0
ASD/PFO	0.005	0.64	0.89
ASD/PFO	0.01	0.52	1.01
ASD/PFO	0.05	0.02	1.12
CM	0.0001	1	0
CM	0.0005	1	0
CM	0.001	0.18	4.97
CM	0.005	0.04	2.05
CM	0.01	0.23	1.23
CM	0.05	0.99	0.84
VSD	0.0001	1	0
VSD	0.0005	0.05	19.45
VSD	0.001	0.15	5.86
VSD	0.005	0.27	1.43
VSD	0.01	0.53	1
VSD	0.05	0.8	0.94

The union of all CHD datasets is considered as well as the most significant subtypes from this analysis. After extracting SNPs in approximate linkage equilibrium (r^2^ < 0.2) from the full set of all common SNPs (see Materials and Methods Section 2.5 for details), for *p*-values ranging from 0.0001 to 0.05, Fisher exact test was performed for the SNPs above and below *p*-value threshold in NB and in the given condition.

**Table 3:** Shared association regions between neuroblastoma and the diverse CHD subtypes.

Disease	Band	pos hg19	SNPs with *p*-value < 10^−3^	Direction of Effect	Lead SNP NB	*p*-value	Lead SNP CHD Subtype	*p*-value
CM	2q35	215590505–215840829	28	opposite	rs3768708	1.09 × 10^−10^	rs34206771	7.15 × 10^−5^
ASD/PFO	12q21.31	85606538–85723868	16	opposite	rs7295242	2.75 × 10^−4^	rs13377665	3.71 × 10^−4^
VSD	12q21.31	85604092–85723868	18	opposite	rs11116772	2.41 × 10^−4^	rs7954427	5.03 × 10^−4^
VSD	3q25.32	157828781–158245883	399	opposite	rs1978779	6.09 × 10^−8^	rs6441201	2.39 × 10^−5^
ToF	14q24.3	79029133–79059667	14	same	rs4643247	5.88 × 10^−5^	rs7159049	7.75 × 10^−5^

For each region is reported the number of SNPs that have an association *p*-value below 10^−3^ in both datasets in that genomic region and the direction of effect, the genomic band, the left and right margins of this region, and its range in bases, and the lead SNPs in NB and in the CHD subtype with association *p*-values.

**Table 4:** Physical distance between lead SNPs in NB and the diverse CHD subtypes.

Band	Disease	Lead SNP	pos hg19	*p*-value	Disease	Lead SNP	pos hg19	*p*-value	Distance	D’	R^2^
3q25.32	NB	rs1978779	158211291	6.09 × 10^−8^	ToF	rs75107964	158458751	1.30 × 10^−7^	247,460	0.7	0.1
4p16.2	NB	rs11944652	4892294	1.61 × 10^−6^	ASD/PFO	rs4689909	4643276	7.75 × 10^−7^	249,018	0.1	0.01

The table shows only cases in which a lead SNP in a susceptibility locus of NB is less than 250,000 bp away from a lead SNP in a susceptibility locus of at least one CHD subtype.

**Table 5:** Regions of spurious colocalization between NB and diverse CHD subtypes.

Disease	Band	pos hg19	Lead SNP NB	*p*-value NB	Lead SNP CHD	*p*-value CHD	PP
CM	6p22.3	21685357–22748186	rs4712656	6.33 × 10^−16^	rs147429944	7.39 × 10^−9^	0.932813
DORV	11p15.4	7436942–8331494	rs204926	6.91 × 10^−12^	rs12807437	1.71 × 10^−3^	0.906466
DORV	2q35	215573795–217714948	rs2070096	3.39 × 10^−11^	rs116515369	2.43 × 10^−4^	0.91838
DORV	3q25.33	157312429–159477493	rs1978779	6.09 × 10^−8^	158680170	8.16 × 10^−7^	0.923649
DORV	4p16.1	8154534–8733618	rs3796727	3.19 × 10^−9^	chr4:8379187:I	5.06 × 10^−3^	0.91279
DORV	6p22.3	21685357–22748186	rs4712656	6.33 × 10^−16^	rs115828798	1.37 × 10^−4^	0.926876
DORV	6q21	103983460–106054975	rs4945714	1.28 × 10^−8^	rs78448955	1.44 × 10^−3^	0.906372
ToF	11p15.4	7436942–8331494	rs204926	6.91 × 10^−12^	rs6578887	3.80 × 10^−5^	0.917802
ToF	2q35	215573795–217714948	rs2070096	3.39 × 10^−11^	rs13023347	5.08 × 10^−5^	0.919856
ToF	3q25.33	157312429–159477493	rs1978779	6.09 × 10^−8^	rs75107964	1.30 × 10^−7^	0.99738

Regions in the table show evidence of association via two distinct variants in NB and in one CHD subtype.

**Table 6:** eQTL mapping performed in shared and colocalizing susceptibility loci.

Gene	Band	Number of SNPs
*BARD1*	2:q35	20
*MFSD1*	3:q25.32	47
*RARRES1*	3:q25.32	49
*RP11-379F4.4*	3:q25.32	47
*RP11-538P18.2*	3:q25.32	12
*RSRC1*	3:q25.32	159
*SHOX2*	3:q25.32	2
*HS.276795*	4:p16.2	4
*MSX1*	4:p16.2	1

Genes whose expression is affected by SNPs in identified susceptibility loci common to NB and CHD (see Results Section 3.4) are shown. Genomic bands and the number of variants analyzed affecting the expression of these genes is also reported.

## Supplementary Materials

The following are available online at www.mdpi.com/xxx/s1, Figure S1: Manhattan plots of neuroblastoma (NB) and different types of congenital heart disease (CHD), Figure S2: Regional association plots of significant loci described in text, Table S1: Characteristics of patients in the neuroblastoma (NB) cohort, Table S2: Evaluation of the extent of shared genetic effects between NB and congenital heart disease (CHD), Table S3: Evaluation of the extent of shared genetic effects between NB and CHD via simulations, Table S4: Physical distance between lead SNPs in NB and the diverse CHD subtypes, Table S5: List of datasets used for epigenetic characterization of significant loci, Table S6: Annotation of epigenetic features in selected SNPs, Table S7: eQTL mapping performed in colocalizing susceptibility loci.
