# Supplementary material for: Exploring Shared Susceptibility between Two Neural Crest Cells Originating Conditions: Neuroblastoma and Congenital Heart Disease
Source: Genes (Basel). 2019 Aug 30;10(9):663. doi: 10.3390/genes10090663 (PMC6771154; doi:10.3390/genes10090663)
Supplement: Supplementary file 1 [file genes-10-00663-s001.zip › genes-511564-suppl/Supplementary_tables_and_figures_captions.docx]

**Figure S1. Manhattan plots of neuroblastoma (NB) and different types of congenital heart disease (CHD)**. From top to bottom: NB, atrial septal defect/patent foramen ovale (ASD-PFO), conotruncal malformations (CM), double outlet right ventricle (DORV), left-sided malformations (LH), transposition of the great arteries (TGA), Tetralogy of Fallot (ToF), ventricular septal defect (VSD). Only *p*-values below 0.05 are shown. Horizontal red line represents the genome wide significance threshold of association (5x10^-8^). Regions described in the text are highlighted in magenta and their genomic locations are reported.

**Figure S2.** **Regional association plots of significant loci described in text**. In blue is NB, in red are different subtypes of CHD.

**Table S1. Characteristics of patients in the neuroblastoma (NB) cohort.** Information about age, international neuroblastoma staging system (INSS) stage, *MYCN* amplification, histology, DNA index and risk stratification is given.

**Table S2. Evaluation of the extent of shared genetic effects between NB and congenital heart disease (CHD).** The union of all CHD datasets is considered as well as specific subtypes from this analysis: atrial septal defect/patent foramen ovale (ASD/PFO), conotruncal malformations (CM), double outlet right ventricle (DORV), left-sided malformations (LH), transposition of the great arteries (TGA), tetralogy of Fallot (ToF) and ventricular septal defect (VSD). After extracting SNPs in approximate linkage equilibrium (r^2^ <0.2) from the full set of all common SNPs (see Materials and Methods section 2.5 for details), for *p*-values ranging from 0.0001 to 0.05, Fisher exact test was performed for the SNPs above and below *p*-value threshold in NB and in the given condition.

**Table S3. Evaluation of the extent of shared genetic effects between NB and CHD via simulations.** The union of all CHD datasets is considered as well as specific subtypes. After extracting SNPs in approximate linkage equilibrium (r^2^ <0.2) from the full set of all common SNPs, empirical *p*-values for the evaluation of shared genetics were computed (see Materials and Methods section 2.5 for details).

**Table S4. Physical distance between lead SNPs in NB and the diverse CHD subtypes.** Lead SNPs were identified if they belonged to a region with at least 10 SNPs within a distance of less than 100 Kb with association *p*-value <10^-5^ (see Materials and Methods section 2.5 for details). Genomic positions and physical distances between two consecutive lead SNPs is reported.

**Table S5. List of datasets used for epigenetic characterization of significant loci.** Columns are: GEO identifier ,cell line, epigenetic marker, and tissue. hNCC: human neural crest cells

**Table S6. Annotation of epigenetic features in selected SNPs.** SNPs selected for the enrichment analysis (see Results section 3.3) are annotated with features from Table S5.

**Table S7. eQTL mapping performed in colocalizing susceptibility loci.** Genes whose expression is affected by SNPs in identified susceptibility loci common to NB and CHD (see Results section 3.4) are shown. Project identifier, sample type, genomic bands and the number of variants analyzed affecting the expression of these genes are also reported.
