# Supplementary figures and images for: Exploring Shared Susceptibility between Two Neural Crest Cells Originating Conditions: Neuroblastoma and Congenital Heart Disease
Source: Genes (Basel). 2019 Aug 30;10(9):663. doi: 10.3390/genes10090663 (PMC6771154; doi:10.3390/genes10090663)

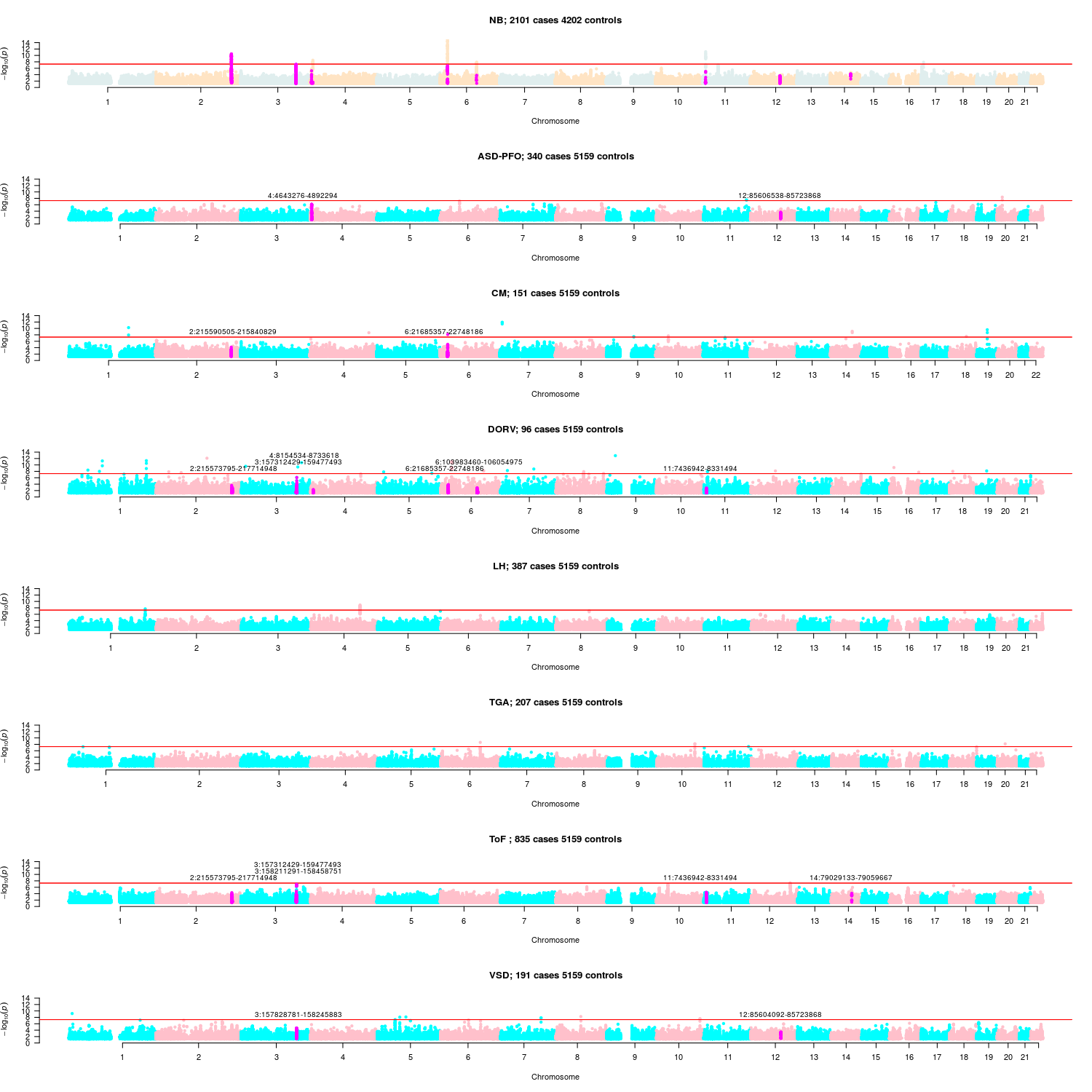

Supplement: Supplementary file 1 [file genes-10-00663-s001.zip › genes-511564-suppl/FigureS1.png]

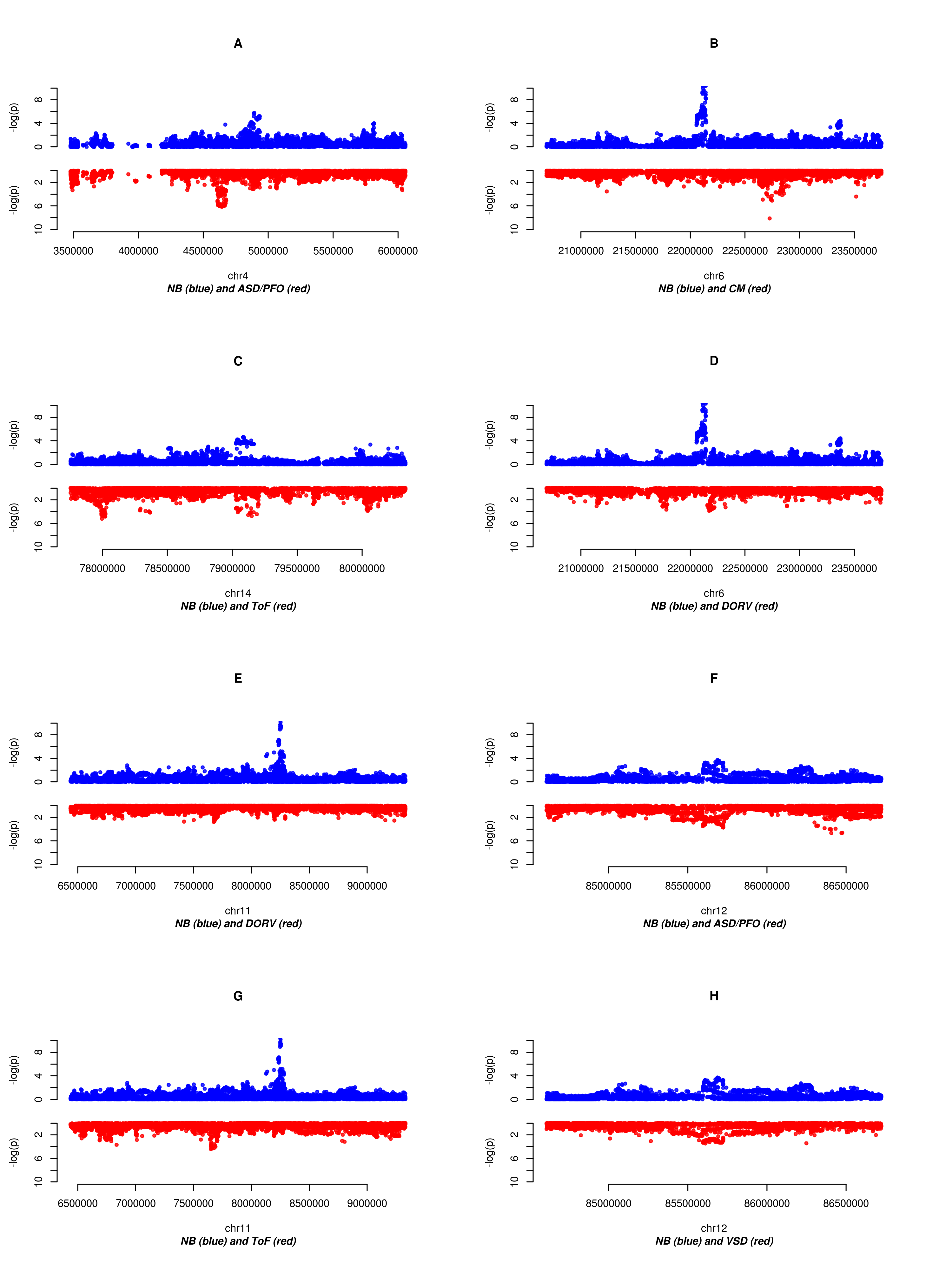

Supplement: Supplementary file 1 [file genes-10-00663-s001.zip › genes-511564-suppl/FigureS2.tiff]
